# Global research trends and prospects on immune-related therapy in ischemic stroke: a bibliometric analysis

**DOI:** 10.3389/fncel.2024.1490607

**Published:** 2024-10-29

**Authors:** Qi Wang, Lei Yuan, Fei Wang, Fei Sun

**Affiliations:** ^1^Medical College, Yangzhou University, Yangzhou, China; ^2^Department of Thoracic Surgery, The Affiliated Taizhou People's Hospital of Nanjing Medical University, Taizhou, China

**Keywords:** ischemic stroke, immune-related therapy, non-neuronal cells, immune response, CiteSpace, bibliometrics

## Abstract

**Background:**

Following ischemic stroke, non-neuronal cells within the nervous system play a crucial role in maintaining neurovascular unit functions, regulating metabolic and inflammatory processes of the nervous system. Investigating the functions and regulation of these cells, particularly immune cells, deepens our understanding of the complex mechanisms of neuroinflammation and immune modulation after ischemic stroke and provides new perspectives and methods for immune-related therapy.

**Methods:**

The annual distribution, journals, authors, countries, institutions, and keywords of articles published between 2015 and 2024 were visualized and analyzed using CiteSpace and other bibliometric tools.

**Results:**

A total of 1,089 relevant articles or reviews were included, demonstrating an overall upward trend; The terms “cerebral ischemia,” “immune response,” “brain ischemia,” “cerebral inflammation,” “neurovascular unit,” and “immune infiltration,” etc. are hot keywords in this field.

**Conclusion:**

In recent years, research on immune-related therapy for ischemic stroke has focused on mechanisms of occurrence, protection and repair of the blood-brain barrier (BBB) by non-neuronal cells, and regulation of immunosuppression and inflammation. Among these, reducing BBB disruption to minimize secondary brain damage has become a hotspot. At the same time, the complex roles of immune responses have attracted attention, particularly the balance between regulatory T cells and Th17 cells in regulating neuroinflammation and promoting neurological function recovery, which is crucial to reduce secondary neuronal damage and improve prognosis, potentially establishing a pivotal frontier in this domain of investigation.

## 1 Introduction

Ischemic stroke (IS) accounts for 75% to 80% of all stroke events, making it the leading cause of cerebrovascular diseases and related deaths worldwide (Cheng et al., 2023). Currently, the preferred treatments for IS are intravenous thrombolysis and arterial embolectomy (Cui et al., 2022). However, these treatment methods are subject to strict time window restrictions, have a narrow range of indications, and are costly, making them inaccessible to many patients (Qiu et al., 2021). In light of this, researchers are tirelessly exploring other effective treatment options.

Following IS, non-neuronal cells within the neural system play a critical role in maintaining the functions of the neurovascular unit, modulating metabolic processes in the nervous system, and mediating inflammatory responses (Liu et al., 2020). Each phase of the inflammatory reaction in the nervous system is intimately associated with specific immune activities, establishing a causal relationship between the two. Both immunity and inflammation are integral components of the ischemic cascade, manifesting as cerebral damage due to arterial occlusion during the acute phase and as tissue reparation in the later stages (Wang et al., 2019). With further research into immune responses activated after IS, immune-related therapy has shown its potential prospects (Iadecola and Anrather, 2011). Therefore, it is particularly crucial to deeply understand the mechanisms of the immune system's response after IS and to further elucidate its complex interactions, thereby exploring new immune-related treatment strategies.

In this study, CiteSpace (Yang et al., 2024) was employed for the pioneering analysis of hotspots and trends on immune-related therapy in IS. The aim is to offer valuable insights for scholars conducting research within this field.

## 2 Materials and methods

### 2.1 Data collection

Web of Science Core Collection (WoSCC) database was chosen as the literature retrieval platform. The retrieval period spanned from 2015 to 2024, with the final search conducted on July 30, 2024. Subject terms were exclusively employed as the search method, and the search formula was: TS = (“Ischemic Stroke” OR “Ischemic Strokes” OR “Ischaemic Stroke” OR “Ischaemic Strokes” OR “Stroke, Ischaemic” OR “Acute Ischemic Stroke” OR “Acute Ischemic Strokes” OR “Ischemic Stroke, Acute” OR “Stroke, Acute Ischemic” OR “Cryptogenic Ischemic Stroke” OR “Cryptogenic Ischemic Strokes” OR “Ischemic Stroke, Cryptogenic” OR “Stroke, Cryptogenic Ischemic” OR “Cryptogenic Embolism Stroke” OR “Cryptogenic Embolism Strokes” OR “Embolism Stroke, Cryptogenic” OR “Stroke, Cryptogenic Embolism” OR “Cryptogenic Stroke” OR “Cryptogenic Strokes” OR “Stroke, Cryptogenic” OR “Wake-up Stroke” OR “Stroke, Wake-up” OR “Wake up Stroke” OR “Wake-up Strokes”) AND TS = (“immu^*^”) AND TS = (“therapy”), document type: Articles or Review Articles; a total of 1,089 documents were retrieved.

### 2.2 Statistical methods

Retrieve the full records and associated bibliographies of the 1,089 documents from WoSCC in Text format, which include 792 articles and 297 reviews. Perform a thorough analysis of the literature using CiteSpace 6.3.R1 (64-bit) Basic, concentrating on the country, institution, authorship, keywords, and cited references. The online bibliometric analysis platform, created by the National Science Library of the Chinese Academy of Sciences, was utilized to undertake a visual examination of historical keywords and international partnerships.

## 3 Results

### 3.1 Annual publication volume in WoSCC

**Figure 1:**
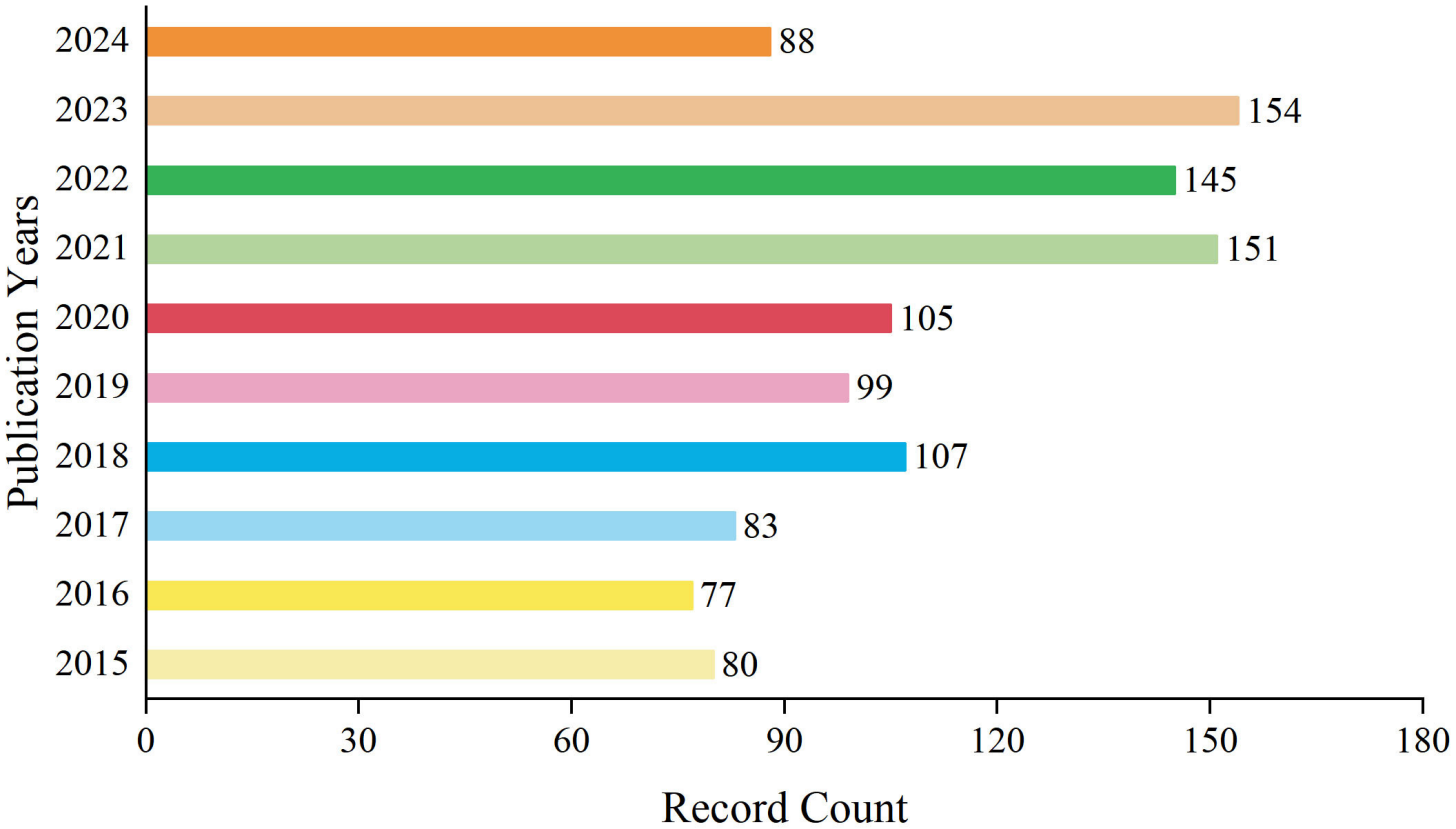
Annual analysis of the number of articles issued.

A total of 1,089 records have been documented over the decade spanning from 2015 to 2024. During this period, the annual publication volume fluctuated. From 2015 to 2017, the number of publications remained relatively low. However, starting from 2018, there was a gradual increase in the publication output, which persisted at a higher level in subsequent years. Notably, in 2021 and 2023, the number of publications peaked at 151 and 154 records respectively, accounting for 13.866 and 14.141% of the total. By 2024, within just over half a year, the number of publications had already reached 88 records, comprising 8.081% of the decade's total. On the whole, the publication trend in this field has exhibited a yearly growth, particularly evident in recent years (Figure 1). This may indicate a heightened interest in research within this domain or an increased investment in research resources.

### 3.2 Distribution of source journals

The literature selected from the 1,089 studies on immune-related therapy in IS have been indexed by 475 journals. For the top 10 journals in terms of publication output, detailed information on Journal Citation Reports (JCR) category, publication quantity, impact factor (IF), and their respective contribution percentages is provided in Table 1.

**Table 1:** Top 10 journals in terms of publication volume.

**Journal titles**	**JCR**	**Number**	**IF**	**Rate%**
Frontiers in Neurology	Q2	30	2.7	2.755
Frontiers in Immunology	Q1	28	5.7	2.571
Journal of Stroke Cerebrovascular Diseases	Q3	23	2.0	2.112
Stroke	Q1	22	7.8	2.02
International Journal of Molecular Sciences	Q1	20	4.9	1.837
Cureus Journal of Medical Science	Q3	15	1.0	1.377
Journal of Neuroinflammation	Q1	15	9.3	1.377
Molecular Neurobiology	Q1	15	4.6	1.377
Plos ONE	Q1	15	2.9	1.377
Neural regeneration research	Q2	14	5.9	1.286

### 3.3 Visualization of collaborations between countries and institutions

**Figure 2:**
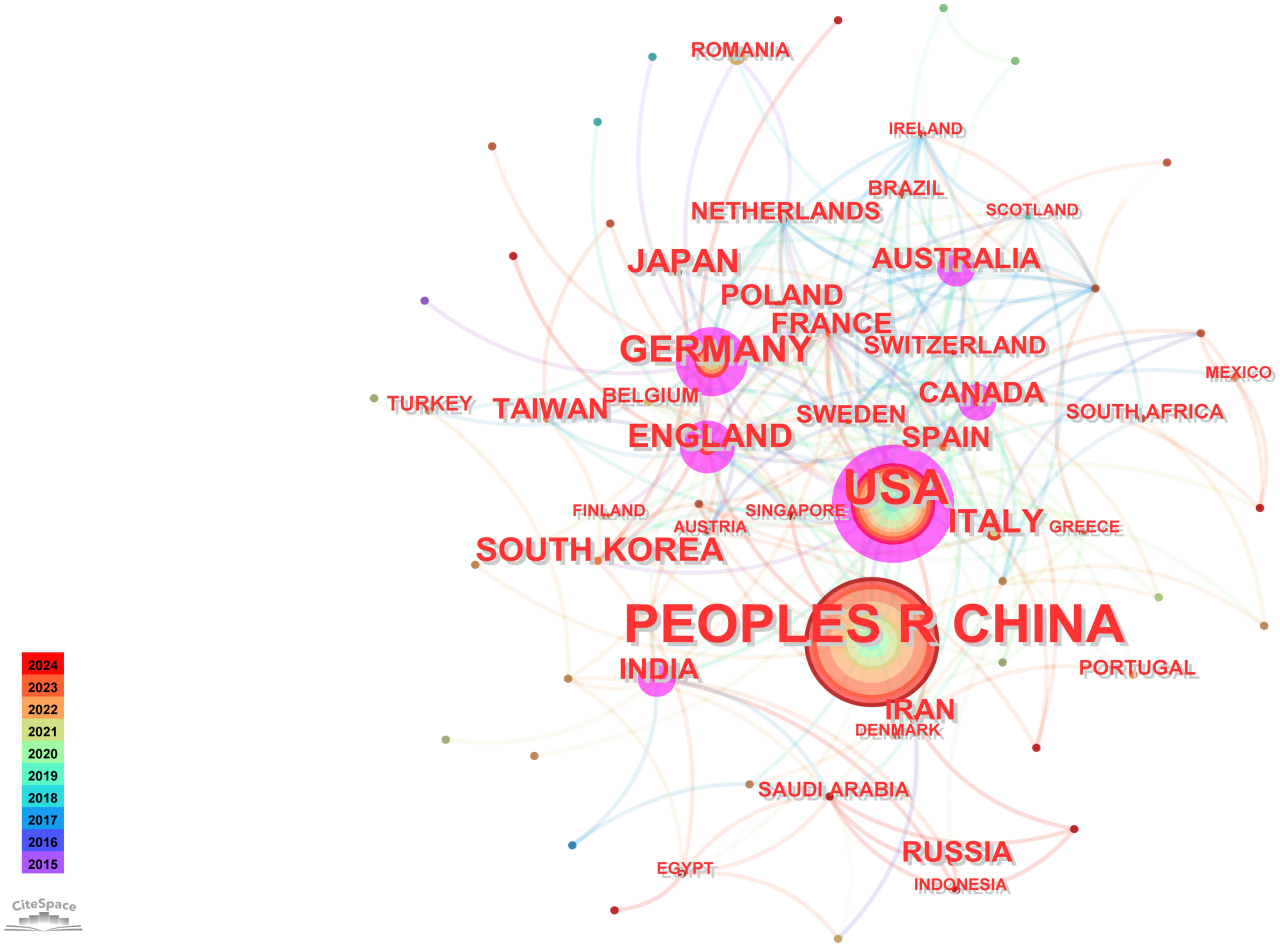
Visual map of countries.

Utilizing CiteSpace software for a country-based analysis yielded a knowledge graph encompassing 74 nodes and 231 links (Figure 2). Each node, represented by a circular shape, signifies a country. The size of these nodes denotes the volume of publications originating from the respective countries. The lines connecting these nodes symbolize collaborative relationships among nations, with the thickness of these connections indicating the intensity of such collaborations. Nodes are color-coded to represent different time periods. Additionally, the size of the purple circles reflects centrality values, which serve as indicators of each country's influence (Chi et al., 2023).

Using the bibliometric online analysis platform (Wang et al., 2023), Figure 3 illustrates the contributions of various countries in the field. Each country's proportional contribution is represented by distinctly colored blocks. Table 2 lists the top five institutions based on their publication output.

**Figure 3:**
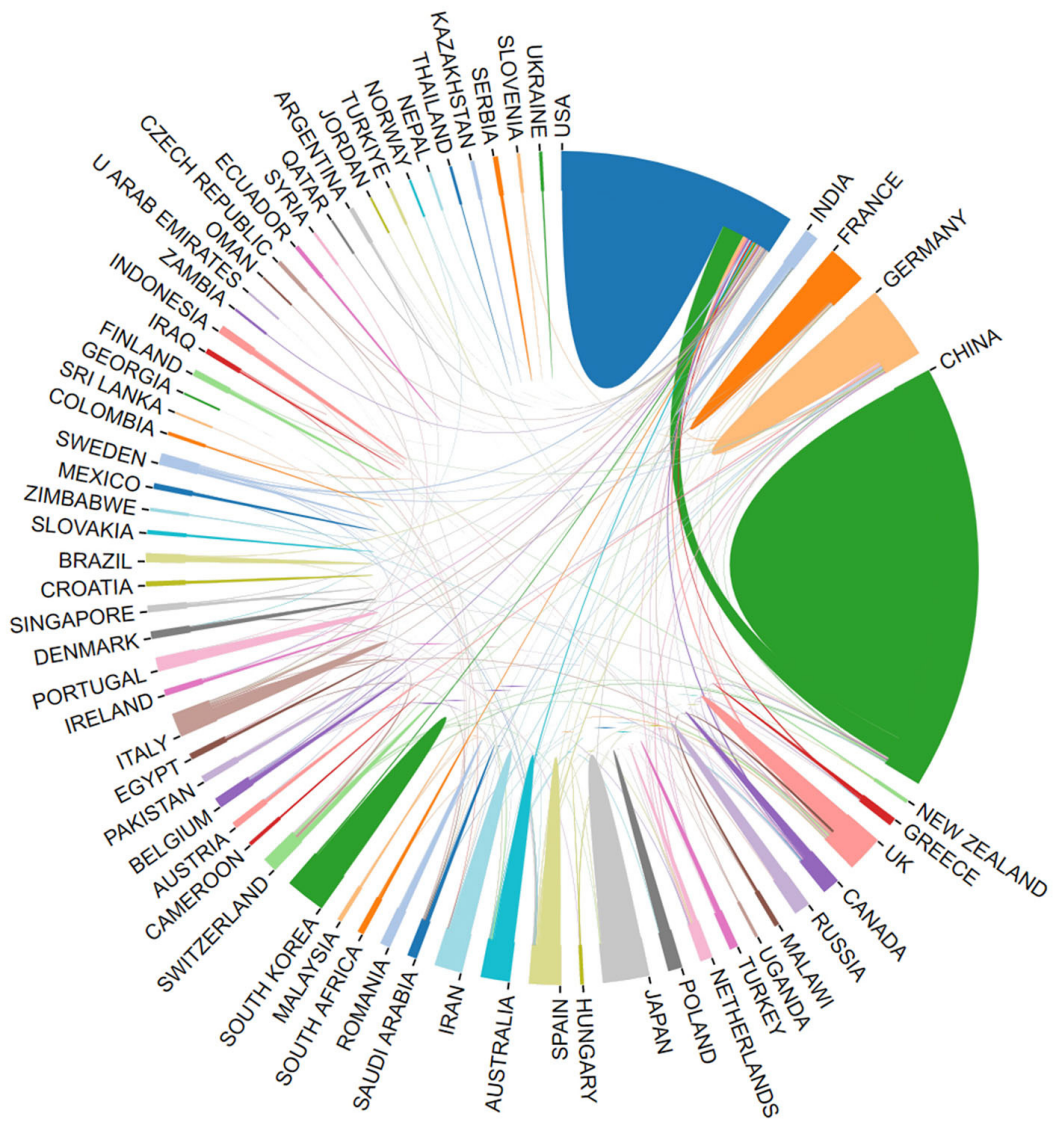
Proportion of national contribution.

**Table 2:** Top five institutions in terms of publication volume.

**Rank**	**Number**	**Institution**	**Country**
1	37	Capital Medical University	China
2	29	Harvard University	USA
3	28	University of California System	USA
4	27	US Department of Veterans Affairs	USA
5	27	Veterans Health Administration VHA	USA

### 3.4 Analysis of author's publication

The findings from the analysis are compiled in Table 3, which lists the top five authors ranked by their publication output. Additionally, the table offers insights into the institutions associated with these authors in relation to the research field. This section provides a quantitative measure of author productivity and identifies leading contributors to the body of research.

**Table 3:** Top five authors in terms of publication volume.

**Rank**	**Author**	**Institution**	**Country**	**Number**
1	Borlongan, Cesar	University of South Florida (USF) College of Medicine	USA	9
2	Xiong, Xiaoxing	Wuhan University	China	8
3	Ji, Xunming	Beihang University	China	8
4	Offner, Halina	Portland VA Medical Center	USA	8
5	Gu, Xiaohuan	Emory University	USA	8

### 3.5 Co-occurrence analysis of keywords

**Figure 4:**
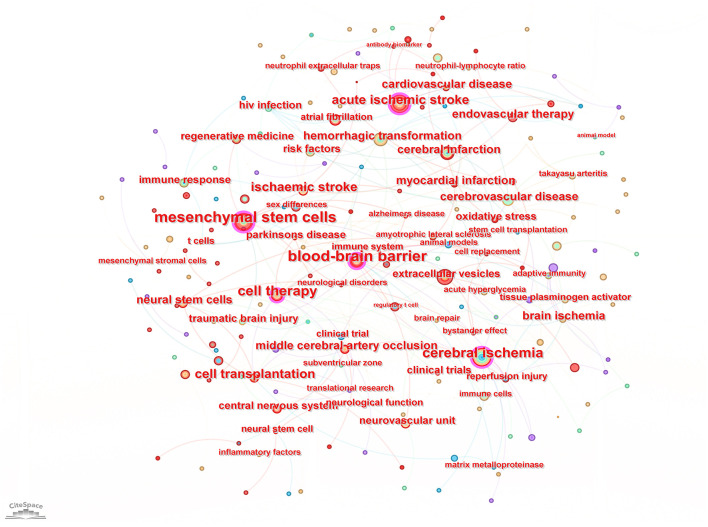
Visual map of author keywords.

**Figure 5:**
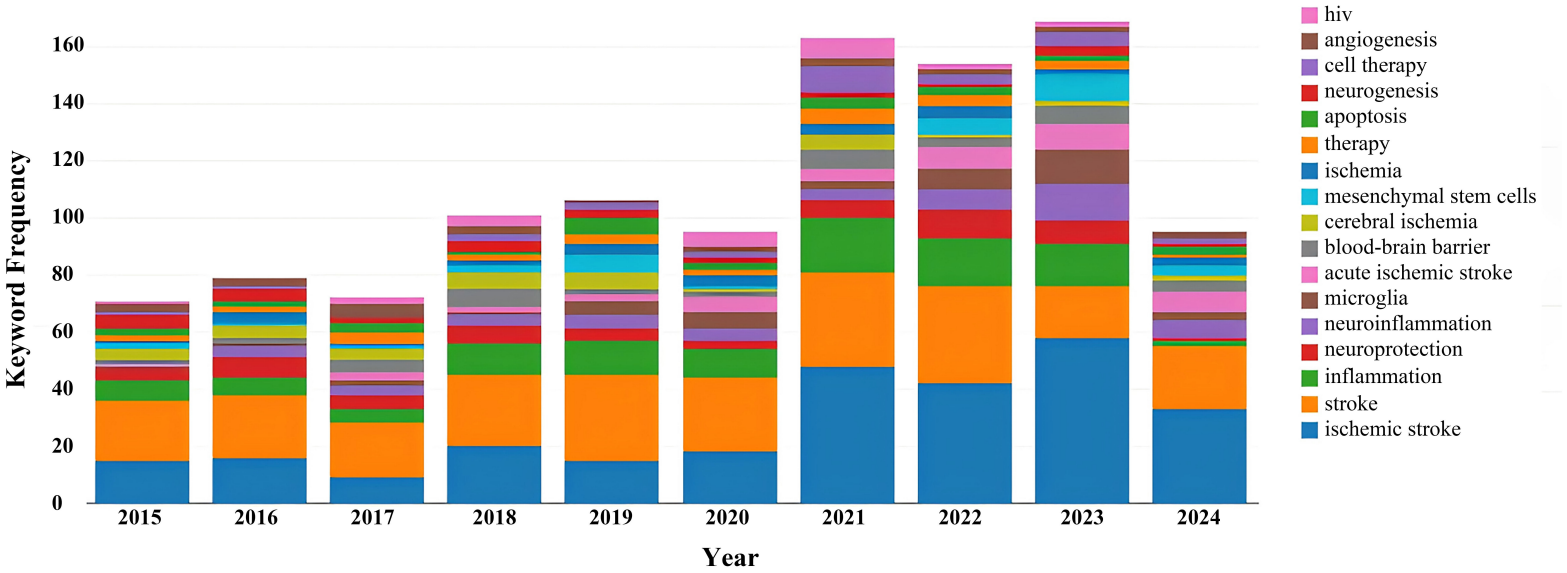
Variation in the number of keywords.

Keyword analysis, particularly through the visualization of co-occurrence patterns, plays a pivotal role in identifying research hotspots and emerging trends in a specific area of study. When the CiteSpace software was employed to analyze author keywords as node types, a keyword co-occurrence network comprising 241 nodes and 352 links was produced (Figure 4). After filtering out search strategy overlaps, an examination of the co-occurrence frequency and centrality metrics of keywords pertinent to this domain (Table 4) disclosed several prominent keywords: blood-brain barrier, mesenchymal stem cells, cell therapy, middle cerebral artery occlusion, extracellular vesicles, immune response, risk factors, clinical trials, regenerative medicine, and regulatory T cells. Figure 5 depicts the evolution of keyword frequencies over time, underscoring the research emphases in recent years concerning immune-related therapies for IS.

**Table 4:** High frequency and centrality keywords.

**Rank**	**Keywords**	**Frequency**	**Keywords**	**Centrality**
1	Blood-brain barrier	37	Cell therapy	0.17
2	Mesenchymal stem cells	31	Blood-brain barrier	0.15
3	Cell therapy	27	Mesenchymal stem cells	0.13
4	Middle cerebral artery occlusion	17	Hemorrhagic transformation	0.10
5	Extracellular vesicles	15	Regulatory T cells	0.06
6	Immune response	13	Middle cerebral artery occlusion	0.05
7	Risk factors	12	Extracellular vesicles	0.05
8	Clinical trials	9	Regenerative medicine	0.03
9	Regenerative medicine	8	Immune cells	0.03
10	Regulatory T cell	4	Immune response	0.02

### 3.6 Keyword burst analysis

**Figure 6:**
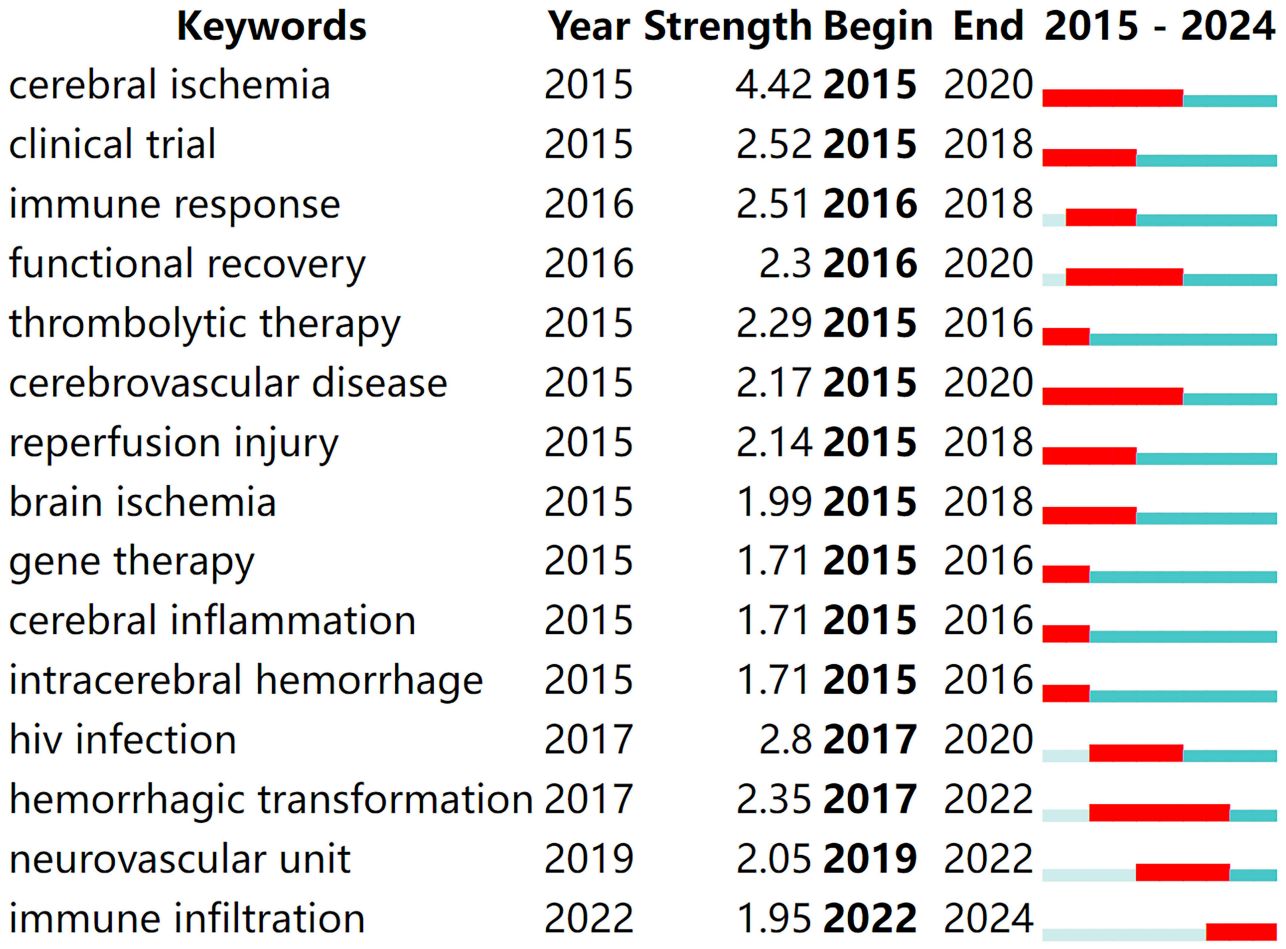
Top 15 keywords with the strongest citation burst.

Keyword burst analysis provides insights into the forefront of research within a given field (Zhou et al., 2023), playing a crucial role in summarizing the recent research directions of immune-related therapies for IS and predicting future hotspots. By running CiteSpace software with a γ-value of 0.7 for burst analysis, the top 15 keywords with the strongest citation bursts were identified, as shown in Figure 6. In the figure, red bands represent a sudden increase in the number of citations for a particular keyword during the corresponding time period. From 2015 to the present, the following terms have emerged as hotspots and cutting-edge areas in the study of immune-related therapies for ischemic stroke: cerebral ischemia, clinical trial, immune response, functional recovery, thrombolytic therapy, cerebrovascular disease, reperfusion injury, brain ischemia, gene therapy, cerebral inflammation, intracerebral hemorrhage, human immunodeficiency virus (HIV) infection, hemorrhagic transformation, neurovascular unit, and immune infiltration.

### 3.7 Cited references

A total of 1,089 pertinent articles were retrieved from the WoSCC, amassing a collective total of 28,292 citations. This results in an average citation count of 25.98 per item. Table 5 presents the top 10 most cited articles within this collection.

**Table 5:** The top 10 cited articles.

**Rank**	**Author**	**Total citations**	**Title**
1	Ma et al.	431	Significance of complement system in ischemic stroke: a comprehensive review (Ma et al., 2019)
2	Letko Khait et al.	420	Wielding the double-edged sword of inflammation: building biomaterial-based strategies for immunomodulation in ischemic stroke treatment (Letko Khait et al., 2021)
3	Makris et al.	396	Blood biomarkers in ischemic stroke: potential role and challenges in clinical practice and research (Makris et al., 2018)
4	Isakovic et al.	381	Mesenchymal stem cell therapy for neurological disorders: the light or the dark side of the force? (Isakovic et al., 2023)
5	Vahidinia et al.	362	Neurosteroids and their receptors in ischemic stroke: from molecular mechanisms to therapeutic opportunities (Vahidinia et al., 2020)
6	Qiu et al.	331	Immune cells in the BBB disruption after acute ischemic stroke: targets for immune therapy? (Qiu et al., 2021)
7	Hu et al.	321	Cerebral vascular disease and neurovascular injury in ischemic stroke (Hu et al., 2017)
8	Si et al.	320	Exosomes in brain diseases: pathogenesis and therapeutic targets (Si et al., 2023)
9	Vahidinia et al.	319	G-protein-coupled receptors and ischemic stroke: a focus on molecular function and therapeutic potential (Vahidinia et al., 2021)
10	Yang et al.	316	A systematic review of the research progress of non-coding RNA in neuroinflammation and immune regulation in cerebral infarction/ischemia-reperfusion injury (Yang et al., 2022)

## 4 Discussion

Research on the pathophysiology of brain damage following ischemic stroke has made considerable progress. Cerebral artery occlusion leads to focal ischemia and infarct development, which in turn results in neuronal injury, necrotic cell death, and secondary inflammatory processes (Javidi and Magnus, 2019). During the early stages of acute ischemic stroke (AIS) development, a robust inflammatory cascade can be observed in the brain. Peripheral immune organs gradually participate in the entire disease process, and BBB begins to be compromised with altered permeability (Jayaraj et al., 2019). The interaction between central and peripheral immunity exacerbates neural and vascular damage, leading to an imbalance in the immune system that increases susceptibility and risk of complications, as well as a poor prognosis (Krishnan and Lawrence, 2019).

In recent years, research trends and hotspots in immunotherapy for ischemic stroke have primarily focused on several key areas: analysis of the mechanisms of occurrence, protection and repair of BBB, and treatments targeting immunosuppression and inflammation. One trend in research is exploring how to reduce secondary brain damage after a stroke by minimizing BBB disruption. Meanwhile, the complex role of immune responses in the progression of ischemic stroke has also garnered significant attention. The balance between immune cells such as regulatory T cells (Tregs) and T helper type 17 (Th17) cells is crucial for controlling post-stroke inflammatory responses and promoting neurological function recovery (Bao et al., 2022). By modulating the activity of these cells, it is possible to reduce secondary neuronal damage and improve prognosis.

### 4.1 Analysis of the mechanisms underlying ischemic stroke

As a multifactorial disease, AIS is influenced by a myriad of genetic and environmental factors, encompassing age, smoking, obesity, atrial fibrillation, hypertension, diabetes mellitus, HIV infection, among others [National Cholesterol Education Program (NCEP) Expert Panel on Detection Evaluation, and Treatment of High Blood Cholesterol in Adults (Adult Treatment Panel III), 2002]. Among these factors, cerebral circulation arteriosclerosis (AS) is one of the significant risk factors leading to stroke occurrence. Patients with AIS often exhibit early vascular aging (EVA), and early monitoring can be achieved through the measurement of pulse wave velocity (PWV). It is noteworthy that smoking, hypertension, and diabetes are significant factors contributing to accelerated arteriosclerosis in this population (Kakaletsis et al., 2024).

In recent years, there has been a sharp increase in the number of stroke hospitalizations among people infected with HIV (Ovbiagele and Nath, 2011). Several clinical studies have confirmed the association between HIV infection and an increased risk of stroke (Alonso et al., 2019; Benjamin et al., 2012; Gutierrez et al., 2017). The specific mechanisms by which HIV infection increases the risk of stroke remain unclear, but it may be due to long-term antiretroviral therapy accelerating arteriosclerosis, thereby increasing the risk of stroke in patients infected with HIV (Benjamin et al., 2012; Donati et al., 2004). Furthermore, various factors associated with HIV infection may increase susceptibility to cerebrovascular disease (CVD), such as opportunistic infections, coagulation abnormalities, dyslipidemia, and the toxicity of antiretroviral therapy. In immunosuppressed patients with HIV infection, sustained viral protein activity can induce tissue inflammation, cellular death, and activation of aberrant signaling pathways. The presence of BBB also restricts drug penetration, allowing the brain to become a reservoir for the virus (Bertrand et al., 2019). Additionally, increased extravasation of neutrophils, microglia, monocytes, and macrophages originating from both cerebral and peripheral infiltration (Lee, 2024) enhances the delay in post-stroke recovery induced by viral overload (Ismael et al., 2020). In the era of antiretroviral therapy, further elucidating the mechanisms by which HIV exacerbates AIS, controlling neuroinflammation to reduce BBB disruption, exploring immune-related therapies, and improving the prognosis of AIS in HIV patients have become new challenges in the treatment of AIS comorbid with HIV.

### 4.2 Repair of the blood-brain barrier and immune infiltration following ischemic stroke

The disruption of BBB is a critical pathophysiological process in acute ischemic stroke (AIS), leading to devastating malignant brain edema and hemorrhagic transformation. The BBB is a unique and tightly regulated anatomical interface between the circulating blood and the central nervous system (CNS), formed by endothelial cells (EC) at the termini of astrocytes and pericytes embedded within the capillary basement membrane (Parvez et al., 2022). The close interaction between ECs, pericytes, astrocytes, microglia, and neurons form the neurovascular unit, which plays a crucial role in maintaining the homeostasis of the CNS neuronal microenvironment through various mechanisms. Under normal physiological conditions, ECs suppress the expression of pro-inflammatory genes and keep circulating leukocytes in a quiescent state (Cheng et al., 2023). Therefore, the blood-brain barrier plays a direct role in regulating immune responses within the central nervous system, rather than acting as a neutral and passive barrier.

The rapid activation of immune cells plays a key role in the disruption of BBB following ischemic stroke. Infiltrating blood-borne immune cells, such as neutrophils, monocytes, and T lymphocytes, can increase BBB permeability as they cause microvascular disturbances and secrete inflammation-related molecules (Zhu et al., 2021). Conversely, they promote BBB repair and angiogenesis in the later stages of ischemic stroke. Accumulating evidence suggests that neuroinflammation plays a dominant role in the progression of brain injury (Shen et al., 2019). Multiple harmful substances, including an excess of cytokine and reactive oxygen species, constitute the stroke-induced inflammatory cascade, leading to compromised vascular integrity, cell death, and secondary brain damage (Iadecola and Anrather, 2011). However, rapid and optimal neuroinflammation is also indispensable in the subsequent processes of injury repair and functional recovery. This dual characteristic may be due to the systemic conditions and time-dependent effects of the immune response (Chu et al., 2015). Given the critical role of immune cells in blood-brain barrier disruption, it is essential to thoroughly explore the inflammatory mechanisms of BBB disruption to identify valuable therapeutic targets for treating ischemic stroke.

In stroke, inflammatory cytokines are significantly increased in the damaged brain regions, local microglia are activated, and the blood-brain barrier is disrupted. Peripheral immune cells, including macrophages, T cells, and B cells, also extensively infiltrate the brain. PD-1 signaling in T cells and B cells, as well as microglia and microphages, participates in post-stroke neuroinflammation (Ren et al., 2011; Bodhankar et al., 2013). Neuroinflammation after ischemic stroke is an important pathological mechanism leading to secondary brain injury, neurodegeneration, and recovery. Although different leukocyte populations are associated with the neuroinflammatory response in stroke, T cells have consistently been shown to be the key cell population driving secondary brain injury. Tregs are an important subset of T cells that secrete immunosuppressive cytokines, maintain immune homeostasis, or limit inflammatory collateral damage by inhibiting autoreactive immune responses (Duffy et al., 2018). Tregs can be classified into several subtypes based on their functions, including CD4+CD25+ Tregs, Tr1 cells, and Th3 cells. Tregs can regulate multiple immune pathways in the pathology of AIS through the secretion of cytokines, cell lysis, and receptor pathways. In addition, Tregs maintain the immune homeostasis by reducing the negative effects of excessive inflammation, preventing cerebral ischemia, and promoting neural repair (Wang et al., 2022). Tregs and Th2 cells can protect the ischemic hemisphere from excessively active immune responses and exacerbate secondary injury progression after AIS. Tregs mainly inhibit the overactivation of resident microglia and infiltrating T cells by upregulating the expression of Interleukin-10 (IL-10) and Transforming growth factor beta (TGF-β), and reduce the levels of pro-inflammatory factors (TNF-α, IFN-g, IL-1β) (Na et al., 2015). Interestingly, Tregs directly inhibit the production of Matrix metallopeptidase 9 (MMP9) in neutrophils by binding to programmed death-ligand 1 (PD-L1) on a transcellular basis, thereby improving BBB integrity and neurological function (Li et al., 2014). Tregs also significantly reduce BBB damage and functional defects by downregulating the expression of endothelial CCL2 and MMP9.

Recent studies have shown that Tregs produce dual-regulated proteins with low affinity for epidermal growth factor receptor (EGFR) ligands to inhibit the proliferation of neurotoxic astrocytes. In addition, pathway analysis has revealed a significant enrichment of pathway genes involved in neuroactive ligand-receptor interactions in Tregs (Ito et al., 2019). Furthermore, related research has also found that increasing Tregs *in vivo* through the JES6-1/IL-2 complex can also reduce neuroinflammatory damage. It confirms that Tregs can promote neurological recovery after IS or prevent recurrent stroke (Tomala et al., 2021). Li et al. (2013) examined the therapeutic potential of Tregs and the mechanisms of neuroprotection *in vivo* in two rodent models of ischemic stroke and *in vitro* in Treg-neutrophil cocultures using a combined approaches including cell-specific depletion, gene knockout mice, and bone marrow chimeras. The results showed that adoptive transfer of Tregs reduced the infiltration of peripheral inflammatory cells into the damaged brain, decreased brain inflammation, and alleviated the integrity of the damaged blood-brain barrier at the early stage of ischemia. A recent study combining single-cell RNA sequencing and flow cytometry methods found that Tregs begin to infiltrate mouse brain tissue 1–5 weeks after experimental stroke. Experimentally selective reduction of Tregs impedes the repair of oligodendrocytes and white matter as well as functional recovery after stroke. Transcriptome analysis indicates that brain-infiltrating Tregs exert effective immunomodulatory effects on monocytes and other immune cells. Additionally, Treg-derived osteopontin enhances microglial repair activity through integrin receptors, thereby promoting oligodendrocyte formation and white matter repair (Shi et al., 2021). Th17 cells correlate with increased cognitive impairment, stroke recurrence, and mortality among AIS patients. MALT1 expression is increased and positively correlates with disease severity, Th1 cells, and Th17 cells, whose high expression severs as an independent risk factor for worse RFS in AIS patients. Th17 cells cause autoimmunity and inflammation, whereas Treg cells inhibit these phenomena and maintain immune homeostasis (Chen et al., 2021; Yu et al., 2022). These results indicate that Tregs play an important role in regulating neuroinflammation and predict that it may be a promising target for IS. In rodent IS models, adoptively transferred Tregs exert protective effects by modulating peripheral neutrophils, thereby preventing proteolytic damage to the BBB. Tregs can play different roles in various stages of IS, so the appropriate timing and complex mechanisms related to Tregs-targeted therapy still require further research.

### 4.3 Post-ischemic stroke peripheral immunosuppression and inflammation

The brain and the peripheral immune system have bidirectional crosstalk. After AIS occurs, BBB is compromised, and stroke-induced immunosuppression followed by infections leads to the infiltration of peripheral immune cells into the damaged brain, which greatly challenges stroke treatment. Although immunomodulation alleviates brain damage in the early stages, it leads to increased immunosuppression and may disrupt repair mechanisms, endangering the brain and worsening long-term outcomes (Ye et al., 2021). The different roles of inflammation at various stages of stroke have drawn attention to the feasibility of treatment plans that completely suppress inflammation. Therefore, a more comprehensive understanding of stroke-induced immunology and inflammation will help develop selective and targeted methods to inhibit their destructive effects.

Most studies on post-stroke immune suppression have focused on the activation of the sympathetic nervous system (SNS). Activation of the SNS triggers the release of catecholamines from the adrenal medulla and sympathetic nerve endings. Then, these catecholamines may act on circulating immune cells that express β2-adrenergic receptors, such as B cells, macrophages, and Th1 cells, inducing apoptosis. However, Th2 cells are not affected, leading to a shift in the global cytokine profile or bias toward a Th2-dominant immune response characterized by the release of IL-10 from activated Tregs, dendritic cells, and M2 monocytes, as well as persistent T cell lymphopenia (Yu et al., 2021). Among them, the hypothalamic-pituitary-adrenal (HPA) axis plays a key role in post-stroke immune suppression. Activation of the HPA axis has also been observed in experimental stroke. Cell factors induced by cerebral ischemia (such as TNF-α, IL-1β, and IL-6) upregulate the synthesis and release of corticotropin releasing hormone (CRH) in the hypothalamus. CRH binds to its receptor and stimulates adrenocorticotropic hormone (ACTH) secretion from the anterior pituitary into the bloodstream (Jin et al., 2013). ACTH then triggers the adrenal gland to produce glucocorticoids. Immune cells express glucocorticoid receptors, and their activation can induce lymphocyte apoptosis. Therefore, activation of the HPA axis can lead to peripheral lymphocyte depletion through the release of glucocorticoids. Studies have shown that Astragaloside IV (ASIV) significantly reduced the levels of cerebral cortex cytokines and CRH expression in the hypothalamus, improved adrenal hypertrophy, and reduced blood corticosterone levels in middle cerebral artery occlusion (MCAO) mice. ASIV did not directly weaken splenocyte apoptosis induced by prednisolone *in vitro*, suggesting that ASIV may improve splenocyte apoptosis through reducing peripheral glucocorticoid levels. In conclusion, ASIV can improve peripheral immune suppression after cerebral ischemia in mice by reducing splenomegaly and lymphocyte depletion, and its potential mechanism involves inhibiting the HPA axis (Wang et al., 2017). The development of post-stroke immune suppression after the initial proinflammatory phase may serve as a protective mechanism to counteract excessive brain damage driven by inflammation. The consequences of post-stroke immune suppression are an increased susceptibility to opportunistic or nosocomial (hospital-acquired) infections, leading to pneumonia and urinary tract infections (Benjamin et al., 2018).

Infections following a stroke may occur within 1 day after the onset of lymphopenia-related immune suppression. Additionally, a reduction in lymphocytes can be observed in patients within 6 h post-stroke, and this condition may persist for at least 1 week or even several months. Infections in patients after a stroke are associated with elevated levels of the anti-inflammatory cytokine IL-10 and decreased levels of the pro-inflammatory cytokine interferon (IFN)-γ in plasma from the first day. This phenomenon involves rapid apoptosis of lymphocytes in the plasma and various lymphoid organs, leading to sustained lymphopenia and post-stroke immune suppression, ultimately increasing the risk of infection. Lymphopenia has also been observed in animal models of stroke (Yu et al., 2021). Post-stroke activation of the sympathetic nervous system (SNS) leads to impaired function of invariant natural killer T (iNKT) cells in the liver, rendering them incapable of fighting off peripheral infections. The bacterial sources of infections following a stroke may originate from aspiration pneumonia or possibly from the translocation of autogenic gut flora through leaky mucous membranes compromised during the stroke event. Studies have found that intestinal inflammation is particularly common in aged mice after a stroke, leading to a higher incidence of spontaneous bacterial infections. Additionally, while macrolide antibiotics might offer some beneficial outcomes for patients with post-stroke infections, most preventative antibiotic treatments fail to improve clinical recovery (Sadarangani et al., 2015).

Furthermore, recent studies have revealed the potential positive role of stem cell transplantation in improving immune regulation after ischemic stroke (IS). Considering the spleen as the primary site for lymphatic drainage, the brain's lymphatic system may serve as an effective pathway for the migration of splenic stem cells to the brain. Human umbilical cord blood (HUCB) cells have been shown to reduce infarct size in rats after permanent middle cerebral artery occlusion (pMCAO), offering protective effects on both gray and white matter, and promoting the recovery of spleen size 48 h post-pMCAO (Seifert and Offner, 2018). Concurrently, studies support the therapeutic potential of targeting peripheral inflammatory responses via the spleen to mitigate neuroinflammation, whereby intravenous administration of human bone marrow stem cells (hBMSC) exhibits preferential migration to the spleen and suppresses systemic inflammation (Xu et al., 2019). Overall, the mechanisms related to post-stroke immune suppression still require further exploration. How to better control peripheral infections triggered by this condition and improve patient outcomes is a direction for future research on immunotherapy.

## 5 Conclusion

This study presents a comprehensive analysis of the literature pertaining to immune-related therapy for IS, as indexed within the WoSCC. The findings offer a preliminary snapshot of contemporary research trajectories and anticipate potential areas of intense scrutiny and innovative advances in upcoming investigations. The objective is to furnish scholars and researchers engaged in the domain of immune-related therapy for IS with meaningful insights and a substantive reference framework.

## Data Availability

The original contributions presented in the study are included in the article/supplementary material, further inquiries can be directed to the corresponding authors.
